# A Comprehensive Review of *Alaria alata* (Goeze 1782) (Platyhelminthes, Trematoda) in Different Animal Hosts

**DOI:** 10.3390/pathogens14070625

**Published:** 2025-06-23

**Authors:** Aneta Bełcik, Tomasz Cencek, Weronika Korpysa-Dzirba, Małgorzata Samorek-Pieróg, Jacek Karamon, Jacek Sroka, Jolanta Zdybel, Marta Skubida, Ewa Bilska-Zając

**Affiliations:** Department of Parasitology and Invasive Diseases, Bee Diseases and Aquatic Animal Diseases, National Veterinary Research Institute/State Research Institute, Partyzantów Avenue 57, 24-100 Puławy, Poland; tcencek@piwet.pulawy.pl (T.C.); weronika.korpysa@piwet.pulawy.pl (W.K.-D.); malgorzata.samorek-pierog@piwet.pulawy.pl (M.S.-P.); j.karamon@piwet.pulawy.pl (J.K.); jacek.sroka@piwet.pulawy.pl (J.S.); j.zdybel@piwet.pulawy.pl (J.Z.); marta.skubida@piwet.pulawy.pl (M.S.); ewa.bilska@piwet.pulawy.pl (E.B.-Z.)

**Keywords:** *Alaria alata*, epidemiology, Parasite life cycle, intermediate hosts, paratenic host, definitive host

## Abstract

This review provides a comprehensive overview of the occurrence of *Alaria alata* (Goeze 1782) trematodes in first, second, definitive, and paratenic hosts, including wild and domestic animals. This systematic review was conducted using two academic databases: Web of Science and Google Scholar. A total of 119 articles containing data on 18 different *A. alata* hosts from 30 countries were analyzed. Based on the literature review, the best-studied group were definitive hosts (Mustelidae, Canidae, and Felidae), followed by paratenic, first (snails), and second intermediate hosts (amphibians). For these key intermediate hosts—snails and frogs—the data remain sparse, highlighting a gap in understanding the possible scale of the spread of *A. alata*. Among definitive hosts, Canids showed a higher prevalence, reinforcing their significant role in the parasite’s spread. Additionally, some Procyonidae, Felidae, and Mustelidae have been identified as paratenic hosts, with mesocercariae localized in their muscle tissues. Considering that meat of unknown origin or meat that is insufficiently heat-treated may contribute to human infection, prevalence rates as high as 40–50% in wild boar highlight the critical need for complex research. Furthermore, this review clarifies the role of host groups in the life cycle and transmission of *A. alata*, providing key epidemiological information and emphasizing the importance of continued research to fill knowledge gaps.

## 1. Introduction

*Alaria alata* trematodes belong to the Diplostomidae family and the genus *Alaria*, with the first report on *A. alata* presented by Goeze in 1782 who found it in the gut of a red fox. These parasites are mainly found in European carnivores. The genus *Alaria* also includes several other species: *A. americana*, (La Rue and Falis 1934) (Paerson 1956), *A. mustelae* (Bosma 1931), *A. intermedia* (Olivier and Odlaug 1938), *A. arisaemoides* (Augustine and Uribe 1927), *A. taxidae* (Swanson and Erickson 1946), and *A. marcianae* (La Rue 1917) [1,2,3,4,5]. It should be noted that the term *A. canis*, introduced by La Rue and Fallis in 1934, is a commonly used synonym for *A. alata*, as explained by Pearson in 1956. These species occur in different geographical locations, mainly in North and South America.

The life cycle of *A. alata* is complex and includes two intermediate hosts, snails (first) and frogs (second), followed by the definitive host (carnivores). This life cycle can be extended through the involvement of paratenic hosts (mainly omnivores and carnivores), which serve as both reservoir and transport hosts [6]. Eggs (oval form, size 110–140 × 70–80 µm) are excreted into the water environment in the feces of the definitive host [6]. After two weeks, the eggs become embryonated, following which miracidia hatch and actively search for snails, the first intermediate host (e.g., the genera *Planorbis*, *Heliosoma*, *Galba* (previously classified within *Lymnaea*), and *Anisus*) [6,7]. Within the first intermediate host, miracidia transform into primary sporocysts, which then reproduce into daughter sporocysts. After the maturation process (lasting approximately one year), the daughter sporocyst starts to produce cercariae forms, with a fork tail. Furcocercariae actively seek and then penetrate the second intermediate hosts, namely frogs, such as the common toads: Bufonidae (*Bufo bufo*, [*B. calamita* = *Epidalea calamita*], [*B. viridis* = *Bufotes viridis*]) or water and brown frogs: Ranidae (*Rana esculenta* = *Pelophylax esculentus*, *R. temporaria*, and *R. arvalis*) and their tadpoles [6,8,9]. In the second intermediate host, furcocercariae are transformed into mesocercariae, which are mainly localized in muscle tissue in frogs (usually tongue muscles, sublingual muscles, and muscles of the limbs) or which live free in the body cavity of tadpoles. Mesocercariae are a larval form (between cercariae and metacercariae) characteristic of *A. alata*. They are oval in form, up to 0.5 mm in length, and have thin parallel lines; they have a mouth aperture and an abdominal sucker. Definitive hosts, e.g., Canidae, Mustelidae, or Felidae, become infected by ingesting a second intermediate host containing mesocercariae. This larval form penetrates the intestinal wall and continues its development during its complex migration through the definitive host’s body [6,10,11,12,13]. After a few weeks in the lungs, it transforms into metacercariae (Diplostomum type). Next, metacercariae migrate to the larynx and are swallowed. Then, they enter the small intestine again, where they develop into the adult form (about 92–114 days after infection). Adult flukes of *A. alata* are 3–6 mm long and about 2 mm wide, with the body divided into two sections. The anterior part of the body has a wing-like shape and ends in the organ of Brandes, which has two functions: parenteral digestion and clinging. The posterior part of the body has a cylindrical form and contains the internal organs. The infections caused by adult forms of *A. alata* in definitive hosts are mostly asymptomatic; however, during acute infection, intestinal inflammation and general poisoning may occur. Paratenic hosts, e.g., wild boars, pigs, rodents, and reptiles (snakes and lizards), also play an important role in the life cycle of this trematode [6,14,15,16,17,18,19]. Mesocercariae can be localized in various parts of their body, mainly in fat and muscle tissue. The parasite does not develop in the paratenic host—the larvae remain in the mesocercariae stage [20,21]. Paratenic hosts are not obligatory for *A. alata* to complete its life cycle, but it does have a significant role in spreading it in the environment.

Humans can also act as a paratenic host for *A. alata*. Infection occurs after consuming raw or semi-raw meat products containing *A. alata* mesocercariae, causing a meat-borne disease known as alariosis [22,23,24]. Symptoms include respiratory disorders, skin problems, headache, fever, runny nose, cough, muscle pain, and inflammation of the retina and optic nerve. In some cases, anaphylactic shock may occur, especially in people with immune system dysfunction [6]. There are several reports in the literature describing clinical cases of *Alaria* spp. infection. The majority of reports come from the United States of America (USA) and Canada [22,25]. These cases resulted from the consumption of *A. alata*-infected frog legs and goose meat that had not undergone appropriate heat treatment [24,26]. The real number of human cases of this zoonosis may be underestimated due to non-specific symptoms reported during the medical interview and difficulties with the diagnosis [27]. Due to the rarity of the disease, the lack of specific serological tests for diagnosing alariosis limits the ability to detect specific antibodies and complicates the monitoring of the disease’s progression and treatment [28,29].

Data on the occurrence of *A. alata* in different animals are variable and depend on the region under study and the type of host examined. The majority of reports on the prevalence of *A. alata* available in the literature usually include occasional findings made during studies covering the diagnosis of other parasites (e.g., *Trichinella* and *Echinococcus*) [30,31].

Therefore, this systematic review was conducted to explore the data on the occurrence of *A. alata* in different animal hosts around the world. We analyzed studies on all the developmental stages of *A. alata* in each possible type of host, taking into account their origin, in order to identify the gaps that need to be filled to obtain a complete picture of the epidemiology of this parasite.

## 2. Materials and Methods

### 2.1. Database Search

The bibliographic data search was performed between 17 and 26 April 2024, using the following websites and databases: Web of Science Core Collection (WoS) (www.webofknowledge.com, accessed on 17 April 2024) and Google Scholar (GS) (https://scholar.google.pl/, accessed on 17 April 2024). The studies included in our review were published between 1961 and the end of 2022.

The literature was screened using the keywords “*Alaria alata*”, which yielded 144 records in WoS and 2530 records in GS, and “*Alaria* spp.”, which returned 126 records in WoS and 6220 records in GS.

After duplicate removal and initial screening, a total of 270 records from databases were assessed. Following title and abstract screening, 134 publications were selected for full-text evaluation. An additional 8750 records were retrieved from other sources (e.g., institutional repositories and other web-based platforms), from which 16 potentially relevant articles were identified.

Following full-text assessment, 119 publications met the inclusion criteria and were included in the final review.

**Figure 1:**
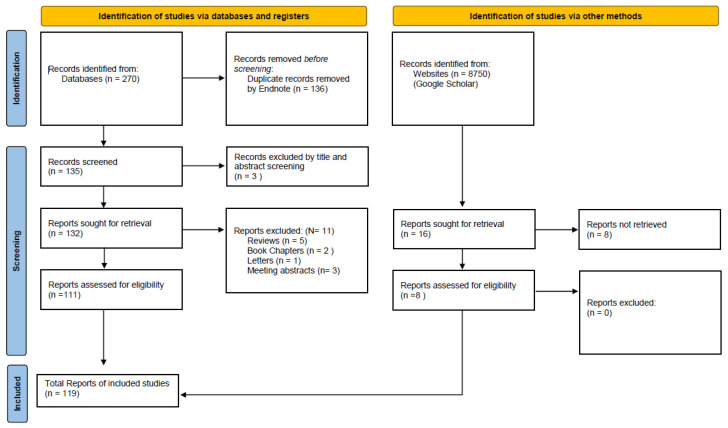
Flow diagram of study selection process and sources of evidence identified from databases and other sources.

The PRISMA 2020 workflow diagram of the database searches and study selection is presented in Figure 1.

### 2.2. Criteria for Data Collection

Articles were reviewed and selected based on the inclusion and exclusion criteria.

A thorough review of the available literature was conducted to assess the frequency of *Alaria alata* occurrence in different host species. Articles that did not include information on frequency or that referred to other *Alaria* species were excluded. The database search also included a review of the available full texts. Where full articles were not available, abstracts were analyzed for relevant information, and abstracts written in English that contained sufficient details on frequency and methodology were included in the review. In cases where full articles were not available in databases, attempts were made to obtain them from other sources, such as institutional repositories, Research Gate, or direct contact with authors.

The preliminary search identified a total of 270 items in online databases such as Web of Science and Google Scholar. After removing 136 duplicate records using End Note, 134 unique items were analyzed based on their titles and abstracts. In addition, 8750 records were obtained from Google Scholar, of which 16 were considered potentially valuable. As a result of the selection process, 23 items were excluded and 111 articles were subjected to full-text evaluation. Of this group, 11 articles were rejected because they were book chapters (*n* = 3), letters to the editor (*n* = 2), or conference abstracts (*n* = 6).

The extracted information was organized according to host species, sample type, diagnostic method used, and the country where the samples were collected.

## 3. Results

### 3.1. Global Distribution of A. alata in Different Countries

Data from 30 countries were collected and included in this systematic review (Table 1). The occurrence of *A. alata* is evident in the geographical distribution of the analyzed studies, with a clear predominance of studies conducted in Europe. The vast majority (over 90%) originated from European countries, specifically Poland (28 articles); Germany (11); Serbia (9); and Belarus, Hungary, and Latvia (7 studies each). In contrast, only a few studies from other continents were identified: North America (Canada and USA, one article each), South America (Brazil and Uruguay, one article each), and Asia (China and Iran, one article each).

### 3.2. Occurrence of A. alata in Different Animal Species

Data on the type of host and the number of articles are summarized in Table 2.

An exploration of the databases revealed data on the occurrence of *A. alata* in each type of host; altogether, 18 different animal species tested positive for this trematode. Two studies described the presence of *A. alata* in first intermediate hosts, specifically two species of snail: *P. planorbis* and *A. vortex* [7,107]. Five articles reported on trematodes in second intermediate hosts: toads (*B. bufo*; *B. calamita*; *B. viridis*) [38] and tadpoles and adult stages of brown frogs sensu lato (e.g., *Rana dalmatina* or *R. temporaria*, *R. esculenta*, and *R. arvalis*) [8,84,107] and water frogs (*Pelophylax ridibundus*, *P. lessonae*, *P. esculentus* complex, and hybrid *P. esculentus*) [8,9,84].

The occurrence of *A. alata* was described in the following definitive hosts: foxes [red fox (*Vulpes vulpes*) pampas fox (*Pseudalopex gymnocercus*), crab-eating fox (*Cerdocyon thous*)], in 38 studies [10,11,12,13,31,37,44,47,50,52,53,56,58,62,63,66,68,69,71,74,75,79,81,83,86,88,89,90,91,93,94,109,113,120,122,129,131,135]; golden jackals (*Canis aureus*), in 4 studies [76,119,120,123]; raccoon dogs (*Nyctereutes procyonoides*), in 13 studies [32,39,52,55,62,66,67,86,94,98,136]; wolves (*Canis lupus*), in 13 studies [46,48,59,60,61,82,83,99,102,109,118,128,130]; dogs (*C. lupus familiaris*), in 9 studies [70,73,77,106,107,124,125,132,134]; wild cats, domestic cats, jungle cats (*F. silvestris*, *F. chaus*, *Felis catus*), in 5 studies [49,78,126,133,134]; Eurasian lynxes (*Lynx lynx*), in 1 study [103]; European otters (*Lutra lutra*), in 2 studies [42,108]; European polecats (*Mustela putorius*), in 1 study [87]; and American minks (*Neogale vison*), in 3 studies [40,87,109].

Finally, studies also included findings on the presence of *A. alata* in the following paratenic hosts: wild boars (*Sus scrofa*), in 26 studies [15,16,17,18,19,33,34,35,36,45,51,65,72,80,85,97,100,101,104,105,110,121,134,137]; pigs (*S. domesticus*), in 1 study [17]; snakes (*Natrix natrix*, *Vipera berus*, and *Coronella austriaca*), in 10 studies [14,41,92,95,96,111,112,114,116,117]; and lizards (*Lacerta agilis*), in 1 study [43]. Potential definitive hosts acting as paratenic hosts were also identified, including raccoons (*Procyon lotor*) (one study) [64], domestic cats (*F. catus*) (one study) [57], Eurasian lynxes (*L. lynx*) (one study) [20], and Eurasian badgers (*Meles meles*) (two studies) [57,100]. Although these species are primarily considered definitive hosts, the presence of *A. alata* mesocercariae in the tongue, mandible, and skeletal muscle tissue suggests that they may have played the role of a paratenic host in the analyzed cases.

### 3.3. Occurrence of A. alata in Different Types of Tissues

Table 3 presents data on the different types of host species included in the analyzed articles, covering the first, second, paratenic, and definitive hosts of *A. alata*, as well as the trematode developmental stage and the method of investigation used. In order to present a comprehensive and representative overview of *A. alata* detection, the focus of this review is on the most frequently applied methods. The methodology used was not specified in the 12 articles.

The detection of the different developmental stages of *A. alata* (eggs, furcocercariae, metacercariae/adult stage, and mesocercariae) largely depends on the type of host, the material submitted for analysis, and the parasitological method selected. However, some authors do not specify the research method and/or the type of sample in their articles. Therefore, several publications are not included in the table.

To organize and systematize the cases of *A. alata* described in the analyzed articles, we have decided to discuss the findings in separate sections for each host type (first intermediate, second intermediate, definitive, and paratenic).

#### 3.3.1. First Intermediate Host of *A. alata*

Data on the prevalence of *A. alata* in the first intermediate host (snails) are summarized in Table 4.

Data describing the occurrence of *A. alata* in common snails were available in two publications, one from Poland and one from France [7,107] (Table 4). The determined prevalence was high in the Polish study, ranging from 29.7% and 42.0% to 100.0% [107], while in the French study, the prevalence was 0.9% in small planorbids (32 infected specimens out of 3431 *P. planorbis* and *A. vortex* tested) [7]. Wójcik et al. [107] collected samples over three different seasons, focusing solely on *P. planorbis*, while Portier et al. [7] collected samples during one season but included additional species: *A. vortex* (a small planorbid), *Planorbarius corn*eus, *Lymnaea stagnalis*, and *Radix* sp.; among them, *A. alata* was only detected in *P. planorbis* and *A. vortex* (small planorbid). Conducting surveys at different times of the year made it possible to determine whether the intensity of invasion changes over time. The highest invasion was recorded in Poland in the spring (100.0%), while the two autumn seasons showed a lower prevalence, at 29.7% and 42.0% [107]. The samples from France were collected during similar seasons (autumn and spring), but the number of samples tested (3431 samples) was significantly higher than that collected in Poland (124, 128, and 600 samples) [107]. Despite the extensive study by Portier et al. [7], the prevalence of *A. alata* furcocercariae was low (0.9%) [7]. This may have been caused by differences in environmental factors and differences in predator–prey interactions in the study area. Variable environmental conditions, such as temperature and/or low humidity, may have impacted the observed prevalence of furcocercariae in snails. Additionally, the methods used in these studies were different (stimulation by providing two hours of continuous lighting and post-mortem examination) and were not fully specified in Wójcik et al. [107].

Considering the relatively low number of reports on *A. alata* in snails (the two studies mentioned above), there is a significant lack of data on the prevalence of this trematode in other regions of the world. The studies from Poland and France yielded diametrically opposing prevalence results, highlighting the variability in the levels of infection obtained and its dependence on environmental and methodological factors. Taking into account that snails are a necessary host for *A. alata* to complete its life cycle, there is a need to create an up-to-date ‘database’ on the prevalence of this parasite in snail populations.

#### 3.3.2. Second Intermediate Host of *A. alata*

Data on the prevalence of *A. alata* in the second intermediate host (toads and brown and water frogs) are summarized in Table 5.

Data describing the occurrence of *A. alata* in its second intermediate hosts included five papers involving surveys of brown frogs (moor frogs, common frogs, and agile frogs, including tadpoles and adults) and water frogs (marsh frogs, pool frogs, edible frogs, and *Pelophylax* species, including tadpoles and adults), as well as one paper on a group of toads (common, natterjack, and European green toads) (Table 5). These studies included investigations of muscle tissue from the head and torso, different organs (lungs, liver, and visceral membranes), forelimbs, hindlimbs, or whole organisms of the collected specimens to detect the second larval stage of *A. alata* and were conducted in Belarus, Latvia, France, Germany, and Poland [8,9,38,84,107] (Table 5). Prevalence in the toad group was diversified and ranged from 8.0 to 67.9%. The prevalence observed in brown frogs was also varied and amounted to 15.8%, 33.3% 54.1%, and 80.0% [8,84,107]. Similarly, the prevalence of *A. alata* recorded in water frogs ranged from 4.3% (tadpoles) in France to 86.7% (adults) in Germany [8,9]. The intensity of invasion in toads ranged from 1 to 1600 parasites per individual. In water and brown frogs, the intensity was significantly lower, with a maximum of 331 parasites per organism.

Certainly, an important aspect of the high prevalence of *A. alata* in frogs and toads is the aquatic environment in which these hosts live and the presence of the eggs and miracidia of this trematode’s first larval stage in that environment. This relationship definitely determined the high intensity of cercariae in toads: up to 1600 larvae were observed in individual amphibians from Belarus [38]. The lower intensity of invasion in brown frogs from Poland (up to 20 parasites) and Latvia (up to 37 parasites) compared to water frogs from France (up to 331 parasites) was probably related to differences in methodology and the type of samples examined. Patrelle et al. [8] used a modified Berman technique, which allows for a complete survey of frogs, which in turn may contribute to more comprehensive and accurate results. On the other hand, the studies from Latvia and Belarus are based on dissection and compression methods, whose accuracy is likely lower, affecting the precision of the results [38,84]. The method used in the Polish study remains unknown, highlighting the need for further research and for the standardization of the methodology used to ensure that results are comparable between different studies [107]. Unifying the methods used would allow for more comprehensive results in frog studies.

Furthermore, the frogs examined by Patrelle et al. [8] came from the same area as the snails studied by Portier et al. [7], confirming the presence of all the necessary elements for *A. alata* to complete its life cycle in this particular region. Notably, observations regarding the localization of furcocercariae in the bodies of frogs indicate that the most common accumulation sites for these parasites are the visceral membranes, internal organs, and muscles of the head region [84]. These predilection sites should be considered when screening frogs for the presence of *A. alata*.

The lack of research on frogs from outside of Europe suggests that further investigation is needed. Extensive screening for the presence of *A. alata* in frogs should be especially conducted in countries where the consumption of raw or undercooked frog legs is the most common, such as the France, Netherlands, Spain, and the USA, and those that have a high export rate of frog legs to other nations, such as Indonesia [143,144,145]. According to the International Union for Conservation of Nature (IUCN), amphibians are the most endangered group among vertebrates. Nevertheless, the European Union does not impose restrictions on their importation [146]. An estimated 4070 tonnes of frogs caught outside the EU are consumed in Europe each year [146]. More accurate monitoring in countries with high import rates of frog legs and countries with high frog production rates is needed to elucidate the real prevalence of *A. alata* in frogs and estimate the potential risks to consumers [146].

#### 3.3.3. Definitive Hosts for *A. alata*

The data on the prevalence of *A. alata* were collected from 14 definitive hosts—red foxes, pampas foxes, crab-eating foxes, raccoon dogs, golden jackals, dogs, wolves, wildcats, jungle cats, domestic cats, Eurasian lynxes, European otters, European polecats, and American minks—representing the most numerous group of species in this review. The full dataset is available in Table S1 (Supplementary Materials).

Data describing the occurrence of *A. alata* in definitive hosts were available in 78 publications from nineteen countries in Europe, three in Asia, and two each in North and South America. The results were analyzed separately for each of the host families (Canidae, Felidae, and Mustelidae). The full dataset is available in Table S1 (Supplementary Materials). The general data are presented in Table 6.

##### Canidae

Foxes (red foxes, pampas foxes, and crab-eating foxes) were the most extensively tested group of definitive hosts, referenced in 38 research papers from 19 countries (Table 6). The majority of these studies (thirty-six) come from Europe; there was only one paper from Asia and one from South America. The majority of the findings were related to the intestines, encompassing 32 relevant papers. The prevalence of trematodes in foxes varied significantly, ranging from 0.1% to 94.8% [10,11,12,13,31,37,44,47,50,52,53,56,58,62,63,66,68,69,71,74,75,79,81,83,86,88,89,90,91,93,94,109,113,120,122,129,131,135]. Specifically, the prevalence of *A. alata* observed in the intestines and feces of foxes in Northern Europe, in countries with a humid climate, was notably high. For example, Estonia reported a prevalence of 90.7% [58], Latvia 87.4% [83], and Lithuania 94.8% [86]. In contrast, Southern European countries, such as Croatia (4.7%) [50], Greece (6.3%) [69], Italy (5.3%) [79], or Spain (ranging from 2.0% to 17.8%) [129,135], characterized by drier climates, exhibited much lower prevalence rates.

Significant differences in the prevalence of *A. alata* were observed not only between different countries but also between different regions of one country. This highlights the influence of local environmental and ecological factors, in addition to macroclimatic ones, on this parasite’s dynamics of transmission. For example, we identified regions where a wide range of *A. alata* has been reported, such as Poland, where rates varied from 1.9% to 93.9% [12,13,31,90,91,93,94,109]. An analysis of the data presented in Table 6 suggests a correlation between humid, water-rich environments and higher *A. alata* prevalence. This observation is further supported by the findings of Balicka-Ramisz et al. [90], who detected *A. alata* in 1.9% of the samples from southern Poland, 2.5% from the southwest, 19.6% in the Lubuskie and Wielkopolskie regions, and as much as 31.6% from the northwest. This pattern is explained by the predominance of lakes and water bodies in the northern and northeastern regions of Poland, contrasting sharply with the much fewer water reservoirs found in the south.

The significant differences in the results obtained in various countries and studies are caused by the different origins of the foxes collected and thus, different climates and environments. Southern Europe, e.g., Croatia [50], is characterized by mountainous, dry areas, probably with fewer infected frogs and snails than countries with wetter climates such as Latvia, Estonia, and Poland [31,58,83]. However, environmental conditions do not always shape the prevalence of the pathogen. In a study by Górski et al. [109] examining the prevalence of *A. alata* in eastern Poland (Białowieża Forest), characterized by a humid climate and with abundant wetlands, a low prevalence of 13.6% was observed. It must be mentioned that the methods used for sample examination may also have an impact on the results. In the reviewed articles, the authors reported many different methods for the investigation of fox intestines and feces (SCT, IST, shaking in a vessel, flotation with sedimentation, flotation with ZnSO_4_, Sheather techniques, washing through a sieve, mucosal scraping, dissection, and macroscopic/histopathological and helminthological examination). These may have resulted in discrepancies in the reported findings.

Several studies reported high percentages of *A. alata* in fox intestines tested using the sedimentation and counting technique (SCT), with prevalence values as high as 87.4%, 90.7%, 93.9%, and 94.8%, which suggests a high potential sensitivity of this method [31,58,83,86]. However, other studies using the same technique demonstrated much lower detection rates (5.3%, 23.9%, 25.6%, 34.4%, and 52%), indicating that the results can vary depending on a number of factors (such as the climate or season of study) [11,52,75,79,122]. In this review, we found four papers with unidentified research methods [71]. For example, the lack of clearly defined methods may have influenced the low prevalence results of *A. alata* in Loos-Frank & Zeyhle [63], who identified the presence of trematodes in only 3 out of 3573 samples (0.1%) when performing a macroscopic examination of the intestines.

Examining different parts of the intestines also has a significant impact on the results obtained. According to Karamon et al. [31], a higher prevalence of *A. alata* was observed in the posterior part of the intestines, which should be taken into account during examination. The intensity of invasion was only reported in 15 papers and ranged from 1 to 2540 parasites per sample [31]. These findings confirm a significant role of foxes in the contamination of the environment with this parasite’s dispersion forms.

Raccoon dogs

Data on the occurrence of *A. alata* metacercariae in raccoon dogs were described in 13 articles from 9 countries (Table 6). The studies examined the intestines, feces, lungs, and digestive tracts of raccoon dogs, revealing a reported prevalence that ranged from 22.2% to 96.5% [32,39,52,55,62,66,67,86,94,98,136]. The highest percentages were found in Lithuania (96.5%) [86], followed by Latvia (83.9%, 49.5%) [83], Germany (71.6%, 44.4%, 40%) [62,66,67], Estonia (68.3%) [136], and Denmark (69.7%, 32.9%) [52,55]. The two studies conducted in Poland found prevalences of 22.2% (juvenile) and 25.6% (adults), as described by Pilarczyk et al. [98], and of 94.3%, in Karamon [94]. The intensity of invasion varied, ranging from 1 to 18,870 parasites per sample, with the highest values reported in Latvia [83]. Considerable differences were observed between Austria and Poland (maximum intensity of 37 and 20 parasites, respectively) [32,98] in comparison to Denmark, Germany, and Latvia (13,305, 16,200, and 18,870 parasites per sample) [52,67,83]. These differences may be attributed to environmental conditions, the availability of intermediate hosts (frogs and snails), and variations in testing methodologies. Sample size also plays a significant role, especially given the variety of diagnostic techniques used, such as the use of whole intestines in the SCT method, in comparison to intestinal scrapings in the IST method.

Golden jackals

Data describing the occurrence of *A. alata* in golden jackals were available from four publications originating from Hungary and Serbia [76,119,120,123] (Table 6). The determined prevalence ranged from 0.9% to 30.0%; however, the highest prevalence was recorded in Serbia, where the flotation technique was applied for fecal testing [120]. However, differences in prevalence can be influenced by the diagnostic method used, as flotation may have limitations in detecting all the parasite stages. The intensity of invasion was reported in one study from Serbia and ranged from 1 to 5 parasites per sample [123].

Compared to foxes and raccoon dogs, the prevalence of *A. alata* in golden jackals is lower. This may result from the geographical range of this species, which maintained an isolated population along the Mediterranean and Black Sea coasts until the mid-20th century. It should be emphasized that the limited number of reports focusing on *A. alata* in golden jackals hinders a comprehensive understanding of the epidemiology and host–parasite interactions of this definitive host. This highlights the need for further research to fill this gap and provide a more robust assessment of the role of golden jackals as hosts of *A. alata* in its expanding geographical range.

Dogs

Data on the occurrence of *A. alata* metacercariae in dogs (domestic and rescue) were described in eight papers from Europe and one from North America (Table 6). The prevalence ranged from 5.0% in Turkey (1 case out of 20 dogs tested) [132] and the USA (9 cases out of 5417 dogs tested, for client-owned dogs) [134] to 50.0% in Hungary (1 positive case) [73]. One article from Hungary reported a single positive case; however, as only one dog was examined, it is difficult to draw any meaningful conclusions [77]. A low prevalence of this parasite was recorded in dogs from southern Europe, e.g., Greece (2.5%) [70] and Turkey (0.2%) [132], as well as dogs from Poland (4.4%, 14.3%) [106,107]. These variations in prevalence could be influenced by the geographical areas of the studies, as different regions have distinct environmental conditions and habitats. The wide range of findings, which may not be directly comparable, may also result from differences in the number of samples tested, further complicating the interpretation of the results. A greater number of samples, such as those reported in Greece (*n* = 281) [70] and Serbia (*n* = 300) [125], generally provide more reliable prevalence estimates, while smaller samples, such as those in studies from Turkey (*n* = 20) [132] and some Polish studies (*n* = 21, *n* = 69) [106,107], may lead to an under- or overestimation of prevalence due to statistical limitations. The intensity of invasion was reported only in papers from Serbia (7–11 parasites) and Turkey (3 parasites in one positive sample) [125,132]; therefore, it is very difficult to assess its impact on the parasite’s spread. The sedimentation and flotation methods were commonly used, but they may vary in their ability to recover parasite eggs or larvae depending on sample preparation. On the other hand, the Baermann technique is more suitable for detecting some larval stages but may not be effective in detecting adult parasites or eggs. This methodological diversity may lead to inconsistent or unreliable results, underscoring the importance of adopting standardized diagnostic methods.

The average prevalence of *A. alata* found in domestic and rescue dogs is still underestimated in many countries due to a lack of studies, which may have an impact on the reported prevalence results. Moreover, specifically in the case of dogs, the majority of the articles analyzed provided data from the 1990s or 2000s; therefore, an update and a review are required.

Wolves

Wolves were the second most frequently tested group of definitive hosts, analyzed in 13 papers (Table 6) from eight countries in Europe and one in North America. The determined prevalence ranged from 0.3% to 92.9% [46,48,59,60,61,82,83,99,102,109,118,128,130]. A high prevalence was recorded in Northern Europe, particularly in Latvia (85.3%, 92.9%) [82,83] and Estonia (88.5%) [59]. We also identified regions with a wide range of *A. alata* prevalence in wolves, e.g., Poland (2.2–80.8%) [99,102,109]. This diversity is probably due to the inclusion of different geographic areas with different environmental conditions, such as wetlands or drylands. These results confirm the correlation between dry areas and low *A. alata* prevalence, as demonstrated by its low frequency (2.2%) in samples taken from wolves in the southern part of Poland (a mountainous region) according to a study by Popiołek et al. [99], and its high frequency in wetlands, with Szafrańska et al. [102] showing a prevalence of 80.8% in wolves from northwestern Poland. Lower percentages were also observed in southern Europe, e.g., in Croatia (0.3%) [48], Spain (2.1%) [128], and Serbia (1.0%) [118]. The intensity of invasion was reported in four studies and ranged from 3 to 5347 parasites per sample. The presence of such a high number of larvae in a single sample suggests a significant parasite density in the environment, increasing the risk of transmission to other intermediate hosts.

Methodology has a significant impact on prevalence results. The SCT method (92.9%) in a study from Latvia [83] and flotation (89.0%; 80.1%) used in studies from Estonia and Poland [59,102] demonstrated the highest detection rates. In contrast, the IST method, used by Ćirović et al. [118], showed a prevalence of only 1.0%, whereas sedimentation/flotation yielded a value of 3.5% in Bindke et al. [60] and 26.3% in Górski et al. [109]. These discrepancies highlight the importance of standardized diagnostic methods for reliable epidemiological assessment.

##### Felidae (Wild Cats, Jungle Cats, Cats, and the Eurasian Lynx)

Wildcats

The prevalence of *A. alata* in wild cats in Croatia was reported by Martinković et al. [49] and amounted to 5.9% (2 positive individuals out of 34 total), as assessed using the flotation method. The intensity of the invasion was not determined. The relatively low prevalence may be influenced by the Mediterranean climate, characterized by hot, dry summers and a limited availability of aquatic environments [49]. These conditions likely reduce the presence of intermediate hosts, such as amphibians (frogs and toads) and snails, which are necessary in the parasite’s life cycle. Moreover, the diet of wild cats, which mainly consists of small mammals, may limit their exposure to infected prey, further contributing to the low prevalence observed.

Jungle cats

The presence of *A. alata* in jungle cats was reported in a study from Iran, with a prevalence of 14.3% [78]. Most records of *A. alata* invasion have been published since the early 2000s, suggesting that the parasite’s presence in various host species has received increased research attention in recent decades. However, data on *A. alata* in jungle cats remain extremely limited, with only a single study confirming its presence. This highlights the scarcity of research on jungle cats as definitive hosts, leaving significant gaps in understanding their role in the parasite’s transmission cycle. Further studies are warranted to assess the true prevalence and ecological significance of *A. alata* in this species, particularly in different geographic regions.

Domestic cats

Data describing the occurrence of *A. alata* in domestic cats were reported in three studies conducted in Spain, Uruguay, and the USA [126,133,134]. The reported prevalence ranged from 0.6% to 25.0%. The intensity of invasion was only recorded in the study from Uruguay, with five helminths [133]. The differences in the number of samples tested in each study (4 samples from Uruguay, 1246 from the USA) and the fact that the cats were studied in various regions—Europe (Spain) and North and South America (USA and Uruguay)—make comparisons between the results challenging, as they show variations due to climate and environmental factors. The sporadic detection of *A. alata* in domestic cats suggests that infections occurring in this species are likely incidental. Furthermore, regular deworming and veterinary care likely reduce invasion risk in domestic cats compared to wild felids. Further research is needed to determine the true epidemiological role of domestic cats as potential definitive hosts of *A. alata*.

Eurasian lynx

The occurrence of *A. alata* in Eurasian lynxes was documented in a single study from Poland, with a reported prevalence of 6.0% (6/100), as determined using the sedimentation and flotation methods [103]. The intensity of invasion ranged from 2 to 15 parasites per individual.

Given the limited number of available studies on Eurasian lynxes, this result should be interpreted with caution and is insufficient to draw general conclusions about the role of this species as a definitive host of *A. alata*. Nevertheless, the infestations observed in lynxes, as well as in domestic, feral, and wild cats, suggest that cats may contribute to parasite transmission both in the wild and in human-associated environments. Further research is needed to assess the significance of felids in the epidemiology of *A. alata* and their role in maintaining its life cycle.

Carnivores often consume first, second, paratenic, or definitive hosts infected with *A. alata* larvae. By preying on these animals, they contribute to the parasite’s life cycle. However, available data indicate that felids are infected with *A. alata* less frequently than species such as foxes. This difference may be attributed to dietary preferences and ecological factors that influence their susceptibility as hosts.

Domestic cats living near human settlements can spread *A. alata* in urban and rural environments, while feral cats and lynxes may facilitate its transmission in natural habitats. Overlapping territories between domestic and wild felids increase the spread of the parasite, making their feeding habits an important factor in *A. alata* transmission between wild and domestic animals.

##### Mustelidae—Definitive Hosts

European otters

Data describing the occurrence of *A. alata* in European otters originated from Belarus and Poland [42,108]. The prevalence was low in both and was estimated at 2.6% and 4.0% in the internal organs and feces tested using coprological and flotation/sedimentation methods [42,108]. The intensity of the invasion was not determined. These findings suggest that while *A. alata* is present in European otters, invasion rates remain relatively low across the studied populations.

European polecats

Data describing the occurrence of *A. alata* in the European polecat were only available from one paper from Lithuania [87]. Nugaraite et al. [87] reported one (12.5%) positive result out of eight specimens examined during the dissection of individual organs. The intensity of the invasion was not determined. Due to the lack of available data on this topic, drawing conclusions about the real role of this host in *A. alata* transmission is difficult. However, it can be inferred that the European polecat does participate in the distribution of *A. alata*, although probably to a lesser extent than, for example, foxes or wolves.

American minks

Data on the occurrence of *A. alata* in American minks were available from three European countries—Belarus, Lithuania, and Poland—and the prevalence ranged from 6.0% to 12.5% [40,87,109]. The intensity of invasion was only determined by Shimalov & Shimalov [40] and amounted to 500 parasites in the tested samples. The low availability of data in the literature makes it difficult to assess the actual role of American mink in the spread of *A. alata*, but the studies do indicate that these animals are certainly a reservoir for this parasite.

#### 3.3.4. Paratenic Hosts of *A. alata*

Data on the prevalence of *A. alata* were recorded in eight paratenic hosts, including wild boars, pigs, snakes, lizards, raccoons, cats, Eurasian lynxes, and badgers, in studies originating from 16 countries. The full dataset is available in Table S2 (Supplementary Materials). The general data are summarized in Table 7.

##### Suidae

Wild boars

In the paratenic host group, 26 papers included findings of *A. alata* in wild boars (Table 7). The majority of the data come from Europe, with one study originating from North America (USA). The prevalence of *A. alata* ranged from 1.0% to 76.7%, with one publication reporting 100.0% prevalence (but this was based on a very limited sample size of two wild boars). The intensity of infection was reported in 13 studies, varying from 1 to a maximum of 908 parasites per sample [15,16,17,18,19,33,34,35,36,45,51,65,72,80,85,97,100,101,104,105,110,121,134,137]. The methods used for parasite detection included AMT, MSM, modified MSM, TRM, and modified digestion with pancreatin bile and pancreatic enzymes.

It was observed that the findings of *A. alata* in wild boars largely depend on the geographic and climate characteristics of the particular areas under investigation. Nine reports from Poland reported a wide prevalence range (from 6.0% to 58.1%) [16,97,100,101,104,105,106,107,110]. The lower detection rates in southern Poland (4.2%, 32.2%) may be due to differences in habitat characteristics or the use of diagnostic methods primarily designed for detecting *Trichinella* nematodes.

The elevation (above sea level) of the analyzed regions also has an impact on the occurrence of *A. alata*. Portier et al. [36] observed a significant decrease in prevalence with simultaneously increasing elevation. Although elevation was not thoroughly analyzed in the study conducted by Ozolina et al. [137] in Latvia, significant regional differences in frequency were noted, ranging from 2.6% in the Ziemeļkurzeme area to 12.8% in the Sēlija region. Moreover, in drier regions such as Southern Europe, lower prevalence rates have been recorded, including 1.0% in Italy and 1.4% in Croatia [51,80].

It has to be mentioned that hunting seasons seem to be correlated with the level of invasion. Higher rates were observed during summer and autumn in comparison to winter. For example, Renteria-Solis et al. [100] reported a prevalence of 7.1% (1/14) in samples collected during autumn–winter (October and December), while Ozolina et al. [137] determined a higher prevalence (43.9%), with a notable increase during summer months.

The diagnostic method used significantly impacts the detection of *A. alata* in the samples analyzed. Ozolina & Deksne (2017) reported a prevalence of 76.7% using the AMT method, while MSM detected only 40.0% positivity in the same sample set [85]. In another study, the same authors observed differences of 43.9% vs. 13.6% depending on whether AMT or MSM was used, respectively [137]. These results underscore the importance of selecting appropriate methods in accurately assessing the prevalence of *A. alata* and the potential for underestimation when less sensitive techniques are used.

Pigs

Only one paper investigating free-range and backyard domestic pigs for the presence *A. alata* was included in this review. The prevalence reported in the study, which originated from Serbia, was low, at 2.8% out of 72 specimens tested [17]. However, this low result may have been due to the method used in the study, i.e., MSM, which is not intended for *A. alata* detection [17]. This limited number of studies highlights the need for more comprehensive research, especially on free-range pigs with access to the natural environment, as they may be more exposed to *A. alata* mesocercariae.

##### Reptilia

Snakes

Data on the occurrence of *A. alata* in snakes were available from 10 articles (Table 7). Muscle tissues or the pericardium were examined using MSM, AMT, post-mortem dissection, and compression methods. The reported prevalence ranged from 4.0% in Romania to 100.0% in Russia and Poland [14,41,92,95,96,111,112,114,116,117]. The high invasion rates of *A. alata* in different snake species (*N. natrix* and *V. berus*) in Poland [14,111,112] and Russia [116,117] are closely associated with their wetland habitats and the presence of first (snails) and second (frogs and tadpoles) intermediate hosts. Additionally, six studies reported the intensity of invasion, with the highest level, documented by Kirilov & Kirilova [116], reaching 1390 flukes found in grass snakes (*N. natrix*). These results highlight the important role of snakes in maintaining the *A. alata* life cycle and facilitating transmission.

Lizards

The occurrence of *A. alata* in lizards was confirmed in a single study from Belarus [43]. The reported prevalence was 17.0%, with an intensity of invasion exceeding 500 mesocercariae per individual [43]. However, the specific methodology used was not described, limiting the reliability and comparability of the findings. The overall lack of data on *A. alata* in lizards highlights the need for further research to clarify their role in the parasite’s life cycle.

##### Procyonidae

Raccoons

This species belongs to the Procyonidae family, which are typically the definitive hosts (carnivores). However, their role as paratenic hosts cannot be excluded, as demonstrated by Renteria-Solis et al. [64], who examined raccoon tongues using AMT and obtained 11 positive results (10.5%) out of 105 samples tested. The detection of mesocercariae suggests that raccoons also function as paratenic hosts. Their dual role underscores the complexity of *A. alata* transmission and the need for further studies to explain the role of Procyonids and other definitive hosts in the parasite’s life cycle.

##### Felidae—Paratenic Hosts

Domestic cats

In a study from Denmark using the AMT method, the occurrence of *A. alata* was confirmed in 3 domestic cats out of 99 tested (3.0%) [57]. The intensity of the invasion was not reported. According to Takeuchi-Storm et al. [57], cats in this context were acting as paratenic hosts due to the fact that the detected larvae were found in tissues and not in the digestive tract. When cats consume infected amphibians or rodents, mesocercariae can accumulate in their tissues without further development, making cats potential reservoirs of this parasite.

Eurasian lynx

The occurrence of *A. alata* mesocercariae in Eurasian lynxes was reported in Latvia by Ozolina et al. [20], with 4 positive cases out of 231 tested. The intensity of invasion reached up to 23 parasites per sample, as determined using MSM and AMT [20]. These results suggest that lynxes may serve as both definitive and paratenic hosts, thus playing a potential role in the maintenance and spread of the parasite in wild carnivore populations.

##### Mustelidae—Paratenic Hosts

Eurasian badgers

*A. alata* was detected in one badger from Poland (100.0%) and six specimens from Denmark (66,7%) [57,100]. According to Renteria-Solis et al. [100], the mean intensity of invasion in the Polish sample was 28 mesocercariae per sample. However, the number of samples tested from both countries was relatively small. Based on the results of muscle tissue investigation, it can be concluded that badgers may act as paratenic hosts for *A. alata*. As carnivores, badgers can acquire mesocercariae by eating infected intermediate hosts, such as amphibians or snails containing larval stages. Further studies with larger sample sizes are necessary to better understand the role of badgers as hosts of *A. alata* and their potential impact on other hosts.

### 3.4. Role of Environmental Factors (Climate and Geography) and Methods of A. alata Detection in First, Second, Definitive, and Paratenic Hosts

Environmental conditions significantly influence the spread of *A. alata*. Wetland areas, which provide abundant first and second intermediate hosts, enhance the likelihood of helminth transmission to both definitive and paratenic hosts. In contrast, regions with dry, mountainous climates exhibit a lower prevalence of this parasite. This is primarily due to a limited food supply and a scarcity of intermediate hosts, resulting in a reduced occurrence of *A. alata*.

The variations in prevalence may stem from the study methodologies employed and the types of materials analyzed. Frequently, these methods were initially designed for detecting other parasites. Most research, particularly involving Canidae, Mustelidae, and Felidae (definitive hosts), as well as paratenic hosts, focused on a broad range of helminths rather than specifically targeting *A. alata*. As a result, *A. alata* was often found incidentally, which may have led to an underestimation of its true presence in these host groups. This underscores the necessity for further studies specifically on *A. alata*, utilizing more precise and sensitive detection techniques. Such efforts would help to accurately assess the role these animal groups play in determining its actual prevalence levels.

The insufficient data on the intensity of invasion in many studies complicates the assessment of the actual risk posed by *A. alata* in various hosts. As a result, the prevalence of *A. alata* may be underestimated, which could lead to a distorted understanding of the parasite’s transmission risk. Additionally, comparing results from different studies that lack information on the intensity of invasion may cloud the true epidemiological picture. Additionally, sampling during spring and summer, which exhibit a higher prevalence compared to winter, is crucial for accurately detecting *A. alata* and understanding its variation within study populations.

Some of the papers reviewed rely on outdated data, necessitating updates to align with the latest findings. Furthermore, the abundance of reports indicating the presence of *A. alata* in wildlife underscores the urgent need for more frequent studies involving domestic animals. Assessing domestic hosts is crucial, as they may significantly contribute to the transmission of this parasite.

This variability in environmental conditions, study seasons, and diagnostic methods poses challenges in comparing prevalence data across different regions. These factors emphasize the importance of designing surveys that consider the habitats of various hosts, climate variations, and the use of appropriately sized samples. By applying these study parameters in future research, we can achieve reliable and comparable results, which is vital for enhancing our understanding of *A. alata* epidemiology and its impact on both animal and human health.

## 4. Conclusions

The data on the occurrence of *A. alata* in both wild and domestic animals, as presented in this review, is generally extensive. However, findings regarding several key intermediate hosts, such as snails and frogs, are notably scarce, indicating a significant gap in the epidemiology of *A. alata*. Consequently, it is essential to gather more detailed and updated information, as these hosts are crucial for the life cycle of this parasite.

In contrast to intermediate hosts, definitive hosts—Mustelidae, Canidae, and Felidae—are better characterized in the reviewed literature. The studies focusing on these groups have yielded more comprehensive results, particularly in terms of prevalence and the intensity of infestation. Notably, Canidae exhibited a higher prevalence of *A. alata* compared to Felidae and Mustelidae, suggesting that this group serves as typical definitive hosts for the parasite, thereby facilitating its widespread distribution.

This review emphasizes the significant prevalence of *A. alata* in paratenic hosts and highlights the need to closely monitor species consumed by humans, particularly wild boars and pigs, due to their association with the risk of alariosis.

Moreover, we observed a lack of data concerning the occurrence of *A. alata* in wild birds within the analyzed studies. The existing literature indicates that cases of alariosis linked to the consumption of goose meat have been documented. Therefore, we must recognize that the widespread availability and high consumption rates of goose and duck meat may pose a genuine risk of alariosis to humans. This situation underscores the critical need for more comprehensive research in this area.

## Figures and Tables

**Table 1:** List of research articles included for analysis in this systematic review.

No.	Country	Number of Articles	Source
1	Austria	7	[10,19,32,33,34,35,36]
2	Belarus	7	[37,38,39,40,41,42,43]
3	Brazil	1	[44]
4	Bulgaria	1	[45]
5	Canada	1	[46]
6	China	1	[47]
7	Croatia	4	[48,49,50,51]
8	Czech Republic	1	[19]
9	Denmark	6	[52,53,54,55,56,57]
10	Estonia	3	[58,59]
11	France	3	[7,8,36]
12	Germany	11	[18,21,60,61,62,63,64,65,66,67,68]
13	Greece	2	[69,70]
14	Hungary	7	[71,72,73,74,75,76,77]
15	Iran	1	[78]
16	Italy	2	[79,80]
17	Ireland	2	[11,81]
18	Latvia	6	[15,20,82,83,84,85]
19	Lithuania	2	[86,87]
20	Netherlands	2	[88,89]
21	Poland	28	[12,13,14,16,31,90,91,92,93,94,95,96,97,98,99,100,101,102,103,104,105,106,107,108,109,110,111,112]
22	Portugal	1	[113]
23	Romania	1	[114]
24	Russia	3	[115,116,117]
25	Serbia	9	[17,118,119,120,121,122,123,124,125]
26	Spain	4	[126,127,128,129]
27	Sweden	1	[130]
28	Turkey	2	[131,132]
29	Uruguay	1	[133]
30	USA	1	[134]

**Table 2:** Summary of host types and total number of publications reporting *A. alata* presence.

No.	Type of Hosts	Host Role	Number of Publications	References
1	Snails	Intermediate host I	2	[7,107]
2	Frogs and toads	Intermediate host II	5	[8,9,38,84,107]
3	Foxes (red, pampas, and crab-eating foxes)	Definitive host	38	[10,11,12,13,31,37,44,47,50,52,53,56,58,62,63,66,68,69,71,74,75,79,81,83,86,88,89,90,91,93,94,109,113,120,122,129,131,135]
4	Raccoon dogs	Definitive host	13	[32,39,52,55,62,66,67,86,94,98,136]
5	Golden jackals	Definitive host	4	[76,119,120,123]
6	Raccoons	Definitive/paratenic host	1	[64]
7	Dogs	Definitive/paratenic host	9	[70,73,77,106,107,124,125,132,134]
8	Wolves	Definitive host	13	[46,48,59,60,61,82,83,99,102,109,118,128,130]
9	Cats (domestic cats, European wild cats, and jungle cats)	Definitive/paratenic host	5	[49,78,126,133,134]
10	European otters	Definitive host	2	[42,108]
11	Eurasian lynxes	Definitive host	2	[20,103]
12	European polecats	Definitive host	1	[87]
13	American minks	Definitive host	3	[40,87,109]
14	Eurasian badgers	Definitive host	2	[57,100]
15	Wild boars	Paratenic host	26	[15,16,17,18,19,33,34,35,36,45,51,65,72,80,85,97,100,101,104,105,110,121,134,137]
16	Pigs	Paratenic host	1	[17]
17	Snakes	Paratenic host	10	[14,41,92,95,96,111,112,114,116,117]
18	Lizards	Paratenic host	1	[43]

**Table 3:** List of methods typically used for detection of all larval stages of *A. alata* in different animal hosts.

Type of Hosts	Type of Sample	Developmental Stage	Method	Source (Example)
First intermediate host - Snails	Whole organism	Furcocercariae	Intravital observation under a light source in a stereomicroscope	[7]
	Hepatopancreas		Post-mortem (microscopically) examination	[107]
Second intermediate hosts (amphibians) - Toads - Brown frogs - Water frogs	Tissue from the head, torso, internal organs (lungs, liver, kidneys, and intestinal wall), visceral membranes, forelimbs, and hindlimbs	Mesocercariae	Baermann technique	[8]
			Dissection with the compression method	[38,84]
			*A. alata* migration technique (AMT)	[9]
Definitive hosts (carnivores) - Canidae - Mustelidae - Felidae	Feces	Egg	Sedimentation/flotation method with ZnSO_4_ (with possible modifications)	[49,131]
			Decantation	[99,102]
			Flotation	[59,76,102,106,107,120,129,134]
			McMaster method according to Raynaud’s protocol	[138]
			Standard sodium acetate–acetic acid–formalin (SAF) technique with ethyl acetate	[139]
			Teleman’s sedimentation	[140]
			Baerman method	[141]
			Modified Wisconsin technique	[46]
			Coprological diagnostics	[82,125]
	Intestines	Adult stage of *A. alata*	Sedimentation and counting technique (SCT)	[11,20,31,52,58,67,74,79,81,83,86,93,94,123,130,136]
			Intestinal scraping technique (IST)	[12,44,53,56,66,88,90,91,118,119,142]
			Shaking in a vessel technique (SVT)	[10,32]
			Mucosal scraping	[89]
			Sheather techniques	[74]
			Post-mortem examination	[13,98]
			Autopsy/microscopic examination	[78]
			Helminthological examination	[82,113,122,135]
	Intestines, feces	Adult stage of *A. alata*	Sedimentation and/or flotation	[55,61,66,103,109,126,132,133]
	Lungs	Metacercariae	Macroscopically and/or histopathologically	[62,82]
Paratenic hosts: - Suidae - Reptilia - Procyonidae - Felidae - Mustelidae	Muscles (diaphragm pillars, peridiaphragmatic adipose tissue, connective tissue from the central tendon of the diaphragm, fat and glandular or muscle, tongue, neck and mandibular, jaw, other skeletal muscles)	Mesocercariae	Magnetic stirrer digestion method (MSM)	[14,15,16,17,20,36,72,85,104,105,112,137]
			Trichinoscopic method (TRM)	[51,97,105]
			*A. alata* migration technique (AMT)	[14,16,18,19,20,33,34,35,45,57,64,65,72,80,85,100,101,105,110,121,134,137]
			Dissection with compression	[41]
			Post-mortem	[92,111]
			Helminthological examination	[43]
			modified digestion with pancreatin bile and pancreatic enzymes (D + P)	[105]

**Table 4:** Occurrence of *A. alata* in snails.

Host	Number of Animals Investigated/Infected/Prevalence (%)	Method of Examination	Season	Type of Sample	Country	Source
*P. planorbis*, *A. vortex* (small planorbid)	3431/32/0.9	stimulated by providing two hours of continuous lighting	from October 2009 to August 2010	whole organism	France	[7]
*P. corneus*	364/0/0.0					
*Lymnea stagnalis*	404/0/0.0					
*Radix* sp.	1336/0/0.0					
*P. planorbis*	600/252/42.0 *	post-mortem examination	autumn 1998 *	hepatopancreas	Poland	[107]
	124/124/100.0 *		spring 1999 *			
	128/38/29.7 *		autumn 1999 *			

* personal communication.

**Table 5:** Occurrence of *A. alata* in toads (*Bufo bufo*, *Epidalea calamita*, and *Bufotes viridis*) and frogs (*Rana arvalis*, *R. dalmatina*, *R. temporaria*; *Pelophylax ridibundus*, *P. lessonae*, hybrid *P*. “*esculentus*”, and *P*. “*esculentus*” complex).

Host	Number of Animals Investigated/Infected/Prevalence (%)	Method of Examination	Type of Sample	Intensity of Invasion/Range	Country	Source
Common toad (*Bufo bufo*)	25/2/8.0	Dissection and compression	Muscle tissue	2–4	Belarus	[38]
Natterjack toad (*Epidalea calamita*)	11/4/36.4			500–1600		
European green toad (*B. viridis*)	28/19/67.9			1–1500		
Moor frog (*Rana arvalis*)	3/1/33.3	Dissection and compression	Head, torso, internal organs (lungs, liver, kidneys, and intestinal wall), visceral membranes, forelimbs, and hindlimbs	2	Latvia	[84]
Common frog (*R. temaporaria*) adults	19/3/15.8			6–37		
Agile frog (*R. dalmatina*) and common frog (*R. temporaria*) tadpoles (two sites)	61/33/54.1	Modified Baermann technique	Whole organism	2–280	France	[8]
Agile frog (*R. dalmatina*) and common frog (*R. temporaria*) adults (two sites)	37/20/54.1			1–331		
Brown frog sensu lato adults	65/52/80.0	Nd *	Tongue, sublingual muscles	10–20	Poland	[107]
Marsh frog (*Pelophylax ridibundus*), pool frog (*P. lessonae*), and edible frog hybrid *P. “esculentus”*	23/1/4.3	Modified Baermann technique	Whole organism	6	France	[8]
Marsh frog (*P. ridibundus*), pool frog (*P. lessonae*), edible frog, and hybrid *P.* “*esculentus*”) adults (two sites)	29/5/17.2			6–314		
*Pelophylax* species adults	15/13/86.7	AMT **	Whole organism	2–20	Germany	[9]
Edible frog (*P. esculentus* complex) tadpoles	80/47/58.8	Dissection and compression	Head, torso, internal organs (lungs, liver, kidneys, and intestinal wall), visceral membranes, forelimbs, and hindlimbs	1–95	Latvia	[84]
Edible frog (*P. esculentus* complex) adults	255/57/22.4			1–237		

* nd, no data (not available); ** AMT, A. alata migration technique.

**Table 6:** Occurrence of *A. alata* in definitive hosts (Canidae, Felidae, and Mustelidae).

Host Species	Total Examined	Total Infected	Prevalence Range (%)	Countries Reported	Source
Canidae					
Red fox (*V. vulpes*)	18,207	4509	0.1–94.8	Austria, Belarus, China, Croatia, Estonia, Denmark, Germany, Greece, Hungary, Ireland, Italy, Latvia, Lithuania, Netherlands, Poland, Portugal, Serbia, Spain, and Turkey	[10,11,12,13,31,37,50,52,54,56,58,62,63,66,68,69,71,74,75,79,81,83,86,88,89,90,91,93,94,109,113,120,122,129,131,135]
Pampas fox (*P. gymnocercus*)	22	8	36.4	Brazil	[44]
Crab-eating fox (*C. thous*)	22	11	50.0	Brazil	[44]
Raccoon dog (*N. procyonoides*)	1764	1144	22.2–96.5	Austria, Belarus, Denmark, Estonia, Germany, Latvia, Lithuania, Poland, and Russia	[32,39,52,55,62,66,67,83,86,94,98,115,136]
Golden jackal (*C. aureus*)	591	29	0.9–30.0	Hungary and Serbia	[76,119,120,123]
Dog (*C. lupus* familiaris)	7008	134	0.2–100.0	Hungary, Greece, Poland, Serbia, Turkey, and USA	[70,73,77,106,107,124,125,132,134]
Wolf (*C. lupus*)	2516	265	0.3–92.9	Canada, Croatia, Estonia, Germany, Latvia, Poland, Spain, Serbia, and Sweden	[46,48,59,60,61,82,83,99,102,109,118,128,130]
Felidae					
European wildcat (*F. s. silvestris*)	34	2	5.9	Croatia	[49]
Jungle cat (*F. chaus*)	7	1	14.3	Iran	[78]
Domestic cat (*F. catus*)	1897	45	0.6–25.0	Spain, Uruguay, and USA	[126,133,134]
Eurasian lynx (*L. lynx*)	100	6	6.0	Poland	[103]
Mustelidae					
European otter (*L. lutra*)	63	2	2.60–4.0	Belarus and Poland	[42,108]
European polecat (*M. putorius*)	8	1	12.5	Lithuania	[87]
American mink (*N. vison*)	89	6	6.0–12.5	Belarus, Lithuania, and Poland	[40,87,109]

**Table 7:** Occurrence of *A. alata* in paratenic hosts (Suidae, Reptilia, Proconidae, Felidae, and Mustelidae).

Host Species	Total Animals Examined	Total Infected	Prevalence (%)	Geographic Range	Source
Suidae					
Wild boars (*S. scrofa*)	40,899	1719	0.6–100.0	Austria, Bulgaria, Czech Republic, Croatia, France, Germany, Hungary, Italy, Latvia, Poland, Serbia, and USA	[15,16,17,18,19,33,34,35,36,45,51,65,72,80,85,97,100,101,104,105,106,107,110,121,134,137]
Domestic pigs (*S. domesticus*)	72	2	2.8	Serbia	[17]
Reptilia					
Snakes (*N. natrix*, *V. berus*, *C. austriaca*)	566	314	4.0–100.0	Belarus, Russia, Poland, and Romania	[14,41,92,95,96,111,112,114,116,117]
Lizards (*L. agilis*)	47	8	17.0	Belarus	[43]
Proconidae					
Raccoon (*P. lotor*)	105	11	10.5	Germany	[64]
Felidae					
Domestic cat (*F. catus*)	99	3	3.0	Denmark	[57]
Eurasian lynx (*L. lynx*)	231	4	1.7	Latvia	[20]
Mustelidae					
Eurasian badger (*M. meles*)	10	7	66.7–100.0	Poland and Denmark	[57,100]

## Data Availability

No new data were created or analyzed in this study. Data sharing is not applicable to this article.

## Supplementary Materials

The following supporting information can be downloaded at https://www.mdpi.com/article/10.3390/pathogens14070625/s1. Table S1. Definitive hosts. Table S2. Paratenic hosts.
