# Supplementary material for: A Comprehensive Review of Alaria alata (Goeze 1782) (Platyhelminthes, Trematoda) in Different Animal Hosts
Source: Pathogens. 2025 Jun 23;14(7):625. doi: 10.3390/pathogens14070625 (PMC12298578; doi:10.3390/pathogens14070625)
Supplement: Supplementary file 1 [file pathogens-14-00625-s001.zip › Supplementary_File_S1.pdf]

Table S1

Host	Number of animals Investigated/infected / prevalence (%)	Method of examination	Type of sample	Intensity of invasion/ range	Country	Source
			Canidae			
Red foxes (*V. vulpes*)	1980/149/7.5	SVT	intestines	nd	Austria	Duscher, 2011
	1213/40/3.3	nd	feces	2-600	Belarus	Shimalov & Shimalov, 2003
	27/3/11.1	flotation	small intestines, feces	17-52	China	Li et al., 2013
	85/4/4.7	IST	small intestines	0-15	Croatia	Rajkovic-Janje et al., 2002
	108/98/90.7	SCT	small intestines	nd	Estonia	Laurimaa et al., 2016
	384/132/34.4	SCT	small intestines	1-1128	Denmark	Al-Sabi et al., 2013
	118/15/12.7 (two sites)	IST	intestines	nd	Denmark	Al-Sabi et al., 2014
	1040/160/15.4	IST	small intestines	nd	Denmark	Saeed et al., 2006
	3573/3/0.1	macroscopic parasite extraction	intestines	nd	Germany	Loos-Frank & Zeyhle, 1982
	470/61/13.0	nd	intestines	nd	Germany	Manke & Stoye, 1998
	79/3/3.8	macroscopically and/or histopathologicall y	lung parenchyma	nd	Germany	Lempp et al., 2017
	80/20/25.0	IST	intestines, feces	nd	Germany	Waindok et al., 2021
	16/1/6.3	zinc sulfate flotation ($ZnSO_4$), merthiolate iodine formaldehyde (MIF) sedimentation	feces	nd	Greece	Liatis et al., 2017
	100/ 48/48.0	Sheather techniques	intestines	0-151	Hungary	Széll et al., 2004
	1612/839/52.0	SCT	intestines	1-20	Hungary	Széll et al., 2013
	68/33/48.5	nd	intestines	nd	Hungary	Andras, 2001
	77/21/27.3	intestinal washing, sieving	intestines	0-308	Ireland	Wolfe et al., 2001
	523/125/23.9	SCT	intestines	1-981	Ireland	Murphy et al.,2012
	57/3/5.3	SCT	intestines	3-4	Italy	Fiocchi et al.,

					2016
539/471/87.4 (adult stage)	SCT	intestines	1-6247	Latvia	Ozolina et al., 2018
468/50/10.7 (metacercariae)	washing, sedimentation	lungs	1-161		
269/255/94.8	SCT	intestines	Nd	Lithuania	Bružinskaitė - Schmidhalter et al., 2011
139/15/10.8	IST	intestines	1-18	Netherlands	Borgsteede, 1984
136/23/16.9	muscosal scraping	intestines	nd	Netherlands	Franssen et al., 2014
639/362/56.7	IST	intestines	nd	Poland	Borecka et al., 2009
22/3/13.6	flotation and sedimentation	feces	nd	Poland	Górski et al., 2006
66/62/93.9	SCT	intestines	113	Poland	Karamon et al., 2016
473/290/61.3	SCT	intestines	1-2540	Poland	Karamon et al., 2018
216/170/78.7	SCT	small intestines	nd	Poland	Karamon et al., 2020
835/264/31.6 (Zachodniopomorskie, Pomorskie provinces)	washing sedimentation	intestines	nd	Poland	Balicka-Ramisz et al., 2003
433/85/19.6 (Lubuskie, Wielkopolskie provinces)					
326/8/2.5 (Dolnośląskie province)					
315/6/1.9 (Śląskie, Opolskie provinces)					
1909/408/21.4	IST	intestines	nd	Poland	Ramisz & Balicka-Ramisz, 2001
620/339/54.7	helminthological examination	small intestine	1-769	Poland	Tylkowska et al., 2018
62/17/27.4	helminthological examination	intestines	nd	Portugal	Eira et al., 2006
172/85/49.4	flotation	feces	nd	Serbia	Ilić et al., 2016
223/57/25.6	SCT	intestines	1-90	Serbia	Miljević et al., 2019
45/8/17.8 (two sites)	nd	intestines	1-42	Spain,Portugal	Segovia et al., 2002
399/8/2.0	helminthological examination	digestive tract	1-42	Spain	Segovia et al., 2004
20/6/30.0	$ZnSO_4$ flotation	intestines	nd	Turkey	Gicik et al.,

						2009
**Pampas foxes (*P. gymnocercus*)**	22/8/36.4	IST	intestines	nd	Brazil	Ruas et al., 2008
**Crab-eating foxes (*C. thous*)**	22/11/50.0	IST	intestines	nd		
**Raccoon dogs (*N. procyonoides*)**	13/3/23.1	SVT	intestines	6-37	Austria	Duscher et al., 2017
	78/37/47.4	helminthological examination	intestines	4-800	Belarus	Shimalov & Shimalov, 2002
	237/78/32.9	sedimentation	feces	nd	Denmark	Kjaer et al., 2021
	99/69/69.7	SCT	intestines	1-13305	Denmark	Al-Sabi et al., 2013
	249/170/68.3	SCT	small intestines	1-200	Estonia	Laurimaa et al., 2016
	74/53/71.6	SCT	intestines	1-16200	Germany	Thiess et al., 2001
	10/4/40.0	macroscopically and/or histopathologicall y	lung parenchyma	nd	Germany	Lempp et al., 2017
	9/4/44.4	IST	intestines, feces	nd	Germany	Waindok et al., 2021
	411/345/83.9 (adult stage)	SCT	intestines	1-18870	Latvia	Ozolina et al., 2018
	333/165/49.5 (metacercariae)	washing, sedimentation	trachea,lungs,	1-592		
	85/82/96.5	SCT	intestines	nd	Lithuania	Bružinskaitė - Schmidhalte r et al., 2011
	53/50/94.3	SCT	intestines	1070	Poland	Karamon et al., 2016
	18/4/22.2 (juvenile raccoon dogs)	post-mortem examination	digestive tract	5-20	Poland	Pilarczyk et al., 2022
	78/20/25.6 (adults raccoon dogs)			6-62		
	17/4/23.5	nd	nd	nd	Russia	Ivanov & Semenova, 2000
**Golden jackals (*C. aureus*)**	20/2/10.0	flotation	feces	nd	Hungary	Takács et al., 2014
	447/4/0.9	IST	stomach and intestine	nd	Serbia	Ćirović et al., 2013
	60/18/30.0	flotation	feces	nd	Serbia	Ilić et al., 2016
	64/5/7.8	SCT	intestines	1-5	Serbia	Miljević et al., 2021
**Dogs (*C. lupus familiaris*)**	2/1/50.0	Baermann technique	feces	nd	Hungary	Majoros et al., 2010
	1/1/100.0	SCT	intestines	nd	Hungary	Szell et al.,

						2001
	281/7/2.5	Teleman's sedimentation method	feces	nd	Greece	Papazahariadou et al., 2007
	69/3/4.4	flotation, decantation	feces	nd	Poland	Wójcik et al., 2001
	21/3/14.3	flotation, decantation	feces	nd	Poland	Wójcik et al., 2002
	60/10/16.7	autopsy	feces	7-11	Serbia	Kulišić et al., 1998
	300/84/28.0	coprological diagnostics methods	feces	nd	Serbia	Marko et al., 2021
	20/1/5.0	sedimentation	feces, intestines	3	Turkey	Umur, 1998
	5417/9/0.2 (client owned dogs)<br>837/15/1.8 (shalter or rescue dog)	flotation	feces	nd	USA	Johnson et al., 2017
**Wolves (*C. lupus*)**	601/93/15.5	modified Wisconsin technique	feces	nd	Canada	Stronen et al., 2011
	400/1/0.3	SAF-technique	feces	nd	Croatia	Hermosilla et al., 2017
	26/23/88.5	flotation	feces	3-1533	Estonia	Moks et al., 2006
	69/11/15.9	sedimentation-flotation and McMaster techniques	feces	nd	Germany	Bindke et al., 2019
	1041/36/3.5	sedimentation-flotation and McMaster techniques	feces	nd	Germany	Bindke et al., 2017
	42/39/92.9	SCT	intestines	7-5347	Latvia	Ozolina et al., 2018
	34/29/85.3	helminthological examination	small intestine, lungs	Nd	Latvia	Bagrade et al., 2009
	89/2/2.2	decantation	feces	nd	Poland	Popiolek et al., 2007
	26/21/80.8 (free-living dogs)	decantation, flotation	feces	nd	Poland	Szafrańska et al, 2010
	19/5/26.3	flotation and sedimentation	feces	nd	Poland	Górski et al., 2006
	47/1/2.1	microscopy	intestines	nd	Spain	Segovia et al., 2001
	102/1/1.0	intestinal parasite examination with flushing and scraping	intestines	4	Serbia	Ćirović et al., 2015
	20/3/15.0	SCT	intestines	5-40	Sweden	Al-Sabi et al.,

						2018
			Felidae			
**wild cats (*F. silvestris*)**	34/2/5.9	flotation with a saturated $ZnSO_4$ solution	feces	nd	Croatia	Martinković et al., 2017
**jungle cats (*F. chaus*)**	7/1/14.3	autopsy, microscopic examination	small intestines	nd	Iran	Tabaripour et al., 2018
**domestic cats (*F. catus*)**	48/1/2.1	coprological methods	small intestines	nd	Spain	Rodriguez-Ponce et al., 2016
	4/1/25.0	flotation, sedimentation	small intestines	0-5	Uruguay	Castro et al., 2009
	1246/17/1.4 (client own cats)	flotation	feces	nd	USA	Johnson et al., 2017
	331/2/0.6 (shelter or resue cats)					
	268/24/9.0 (feral cats)					
**Eurasian lynxes (*L. lynx*)**	100/6/6.0	sedimentation, flotation	feces	2-15	Poland	Szczęsna et al., 2008
			Mustelidae			
**European otters (*L. lutra*)**	25/1/4.0	coprological methods	internal organs	nd	Belarus	Shimalov et al., 2000
	38/1/2.6	flotation, sedimentation	feces	nd	Poland	Górski et al., 2021
**European polecats (*M. putorius*)**	8/1/12.5 (wetlands area)	dissection of individual organs	gastrointestin al tract	nd	Lithuania	Nugaraitė et al., 2019
**American minks (*N. vison*)**	50/3/6.0	dissection	nd	500	Belarus	Shimalov & Shimalov, 2001
	39/3/7.7 (wetlands area)	autopsy/microsco pic examination	gastrointestin al tract	nd	Lithuania	Nugaraitė et al., 2019
	16/2/12.5	flotation and sedimentation	feces	nd	Poland	Górski et al. 2006

nd - no data (not available), AMT - A. alata migration technique, IST - intestinal scraping technique, SCT - sedimentation and counting technique, SVT - Shaking in a vessel technique, SAF - technique- standard sodium acetate acetic acid formalin, helminthological examination – included: macroscopic inspection, dissection of relevant tissues, and microscopic identification of larvae and/or adult helminths
