# Supplementary material for: A Comprehensive Review of Alaria alata (Goeze 1782) (Platyhelminthes, Trematoda) in Different Animal Hosts
Source: Pathogens. 2025 Jun 23;14(7):625. doi: 10.3390/pathogens14070625 (PMC12298578; doi:10.3390/pathogens14070625)
Supplement: Supplementary file 1 [file pathogens-14-00625-s001.zip › Supplementary_File_S2.pdf]

Table S2

Host	Number of animals Investigated/ infected/ prevalence (%)	Method of examination	Type of sample	Intensity of invasion/ range	Country	Source
			**Suidae**			
**wild boars (*S. scrofa*)**	1014/44/4.3	AMT	fat and glandular or muscle tissue (diaphragm or cheek muscles)	nd	Austria	Sailer et al., 2012
	451/30/6.7	AMT	fat and glandular or muscle tissue (diaphragm or cheek muscles)	nd	Austria	Paulsen et al., 2012
	348/21/6.0	AMT	fat and glandular or muscle tissue from cheek	1-47	Austria	Paulsen et al., 2014
	2/2/100.0	AMT	diaphragm pillars	1	Bulgaria	Riehn et al., 2014
	221/15/6.8	AMT	adipose and glandular tissue and muscle	3-69	Czech Republic	Paulsen et al., 2013
	210/3/1.4	TRM	muscle tissue	nd	Croatia	Jaksic et al., 2002
	27582/169/0.6	MSM	diaphragm pillars chiefly tongue,	nd	France	Portier, J., 2014
	286/33/11.5	AMT	diaphragm pillars, tongue, mix of these tissues	2-120	Germany	Riehn et al., 2012
	354/100/28.2	AMT	tongue, abdominal fat tissue	1-908	Germany	Kästner et al., 2021
	315/5/1.6	AMT, MSM	salivary gland, subcutaneous fat and	1-7	Hungary	Berger & Paulsen, 2014

		muscle tissue			
100/1/1.0	AMT	diaphragm pillars	1	Italy	Gazzonis et al., 2018
60/46/76.7	AMT	diaphragm pillars, tongue and other skeletal muscles	1-61	Latvia	Ozolina & Deksne, 2017
60/24/40.0	MSM		1-28		
1233/101/8.2	MSM	diaphragm pillars	1-229	Latvia	Berge & Keidane, 2014
485/213/43.9	AMT	diaphragm pillars, tongue, skeletal muscles	nd	Latvia	Ozolina et al., 2020
485/66/13.6	MSM				
18/8/44.4	nd	diaphragm pillars, dorsal mandibular muscle	nd	Poland	Wójcik et al., 2001
62/13/21.0	modified MSM	muscle tissue	nd	Poland	Wójcik et al., 2002
83/5/6.0	TRM	diaphragm pillars	nd	Poland	Michalski & Wiszniewska-Łaszczych, 2016
3589/151/4.2	AMT, MSM	diaphragm pillars, pharynx area, muscle tissue, connective, adipose, glandular, lymphatic tissues	1- 21	Poland	Bilska-Zając et al. 2021
221/98/44.3	AMT	diaphragm pillars, peridiaphragmatic adipose tissue, connective tissue from the central tendon of the diaphragm	1-79	Poland	Strokowska et.al., 2020
232/104/44.8	AMT	adipose and peritoneal tissue	nd	Poland	Strokowska et.al., 2021a

	43/20/46.5	MSM				
	43/25/58.1	modified digestion with pancreatin bile and pancreatic enzymes (D + P)				
	19/7/36.8	TRM				
	500/161/32.2	MSM	diaphragm pillars	1-22	Poland	Strokowska et al., 2021b
	576/212*/36.8*	AMT	muscle tissue, adipose, connective tissue	nd	Poland	Klich et al., 2022
	14/1/7.1	AMT	tongue, neck, and mandibular	10	Poland	Rentería-Solís et al., 2018
	590/61/10.3	AMT	diaphragm pillars, adipose/connective tissue from neck/ventral cheek	1-26	Serbia	Malesevic et al., 2016
	200/6/3.0	MSM	diaphragm pillars	nd	Serbia	Gavrilović et al., 2019
	43/11/25.6	AMT	tongue, jowl	nd	USA	Johnson et al., 2017
**Domestic pigs (*S. domesticus*)**	72/2/2.8 (free-range and backyard)	MSM	diaphragm pillars	nd	Serbia	Gavrilović et al., 2019
			**Reptilia**			
**Snakes (*N. natrix*, *V. berus*, *C. austriaca*)**	93/20/21.5	dissection, compression	muscle tissue	>500	Belarus	Shimalov & Shimalov, 2000
	25/1/4.0	nd	muscle tissue	72	Romania	Mihalca et al., 2007
	91/90/98.9 (*N. natrix*)	nd	muscle tissue	3-1390	Russia	Kirillov & Kirillova, 2021
	25/12/48.0 (*V. berus*)			1-613		

	27/27/100.0 (*N. natrix*)	nd	muscle tissue	2-18	Russia	Romashow & Romashova, 2022
	7/7/100.0 (*V. berus*)			2-627		
	12/8/66.7	post-mortem	muscle tissue	nd	Poland	Grabda-Kazubska, 1961
	7/7/100.0	post-mortem	pericardium	nd	Poland	Sulgostowska, 1971
	62/29/46.8	nd	muscle tissue	nd	Poland	Lewin, 1992,
	152/70/46.1	nd	muscle tissue	nd	Poland	Lewin, Grabda-Kazubska, 1997
	16/13/81.3	MSM	muscle tissue	3-45	Poland	Zając et al., 2016
	51/30/58.8	AMT, MSM	muscle tissue	13-366	Poland	Bełcik et al., 2022
**Lizards (*L. agilis*)**	47/8/17.0	helminthological examination	muscle tissue	>500	Belarus	Shimalov et al., 2000
			Procyonidae			
**Raccoons (*P. lotor*)**	105/11/10.5	AMT	tongue	nd	Germany	Renteria-Solis et al.,2013
			Felidae			
**Domestic cats (*F. catus*)**	99/3/3.0	AMT	muscle tissue	nd	Denmark	Takeuchi-Storm et al., 2015
**Eurasian lynx (*L. lynx*)**	231/4/1.7	AMT, MSM	muscle tissue	23	Latvia	Ozolina et al., 2020
			Mustelidae			
**Badgers (*M. meles*)**	1/1/100.0	AMT	tongue, neck, mandibular	28	Poland	Rentería-Solís et al., 2018
	9/6/66.7	AMT	muscle tissue	nd	Denmark	Takeuchi-Storm et al., 2015

**- personal communication, nd - no data (not available), AMT - A. alata migration technique, MSM – magnetic stirrer (digestion method), TRM - trichinoscopic method, helminthological examination – included: macroscopic inspection, dissection of relevant tissues, and microscopic identification of larvae and/or adult helminths*
